# Improving Willingness to Try Fruits and Vegetables and Gross Motor Skills in Preschool Children in Guam

**DOI:** 10.3390/nu14010093

**Published:** 2021-12-27

**Authors:** Tanisha F. Aflague, Grazyna Badowski, Hyett Sanchez, Dwight Sablan, Catherine M. Schroeder, Eloise Sanchez, Rachael T. Leon Guerrero

**Affiliations:** 1 College of Natural and Applied Sciences, University of Guam, Mangilao, GU 96923, USA; gbadowski@triton.uog.edu (G.B.); sanchezh@triton.uog.edu (H.S.); 2 Office of Research & Sponsored Programs, University of Guam, Mangilao, GU 96923, USA; sabland9706@triton.uog.edu (D.S.); rachaeltlg@triton.uog.edu (R.T.L.G.); 3 Guam Head Start Program, Guam Department of Education, Barrigada, GU 96913, USA; cmschroeder@gdoe.net; 4 Division of Curriculum & Instruction, Guam Department of Education, Barrigada, GU 96913, USA; esanchez@gdoe.net

**Keywords:** preschool children, motor skills, fruit and vegetable intake, Guam

## Abstract

Early childhood interventions have the potential to promote long-term healthy eating and physical activity habits to prevent obesity. However, research studies including indigenous young children are lacking. This study examined the effectiveness of the Food Friends^®^: Fun with New Foods™ and Get Movin’ with Mighty Moves™ (FFMM) curricula on willingness to try fruits and vegetables (FV) and gross motor (GM) skills among preschoolers in Guam. A pre-post community-based study included preschoolers from Head Start (HS), gifted and talented education (Pre-GATE), and Pre-Kindergarten programs during school years (SY) 2017–2018 and 2018–2019. In SY2017–2018, the intervention group had a significant increase in imported FV when compared with the other three groups. No significant differences between groups were found on the other FV scales. Regarding gross motor skills, no significant differences between groups were found. In SY2018–2019, the intervention group had a significant increase in all FV scales except imported FV when compared with the enhanced intervention group. With gross motor skills, no significant differences were found between groups on its progress. These results warrant FFMM adaptations for the prevention of obesity among Guam preschoolers.

## 1. Introduction

Early childhood overweight and obesity (OWOB) increases the risk for adult OWOB and associated chronic diseases, which are high among indigenous children [1]. The prevalence of early childhood overweight and obesity (OWOB) in 2011–2012 was 22.8% among 2–5 years old in the US [2]. In 2013, the OWOB prevalence among children 2–8 years old in Guam was 27.4% [3]. Early childhood obesity prevalence was also higher in Guam (13.2%) than the US (8.4%) during the same time and among similar ages [2,3]. Successful obesity interventions for young indigenous and/or socioeconomically disadvantaged children (0–5 years), employed a dual focus on obesity prevention and school readiness, engaged children and parents in educational activities related to nutrition and physical activity, and physical activity sessions that focused on the development of gross motor skills [4]. 

A recent community randomized environmental childhood obesity intervention in Guam and other jurisdictions in the US Affiliated Pacific region, known as the Children’s Healthy Living (CHL) project, found a decrease in OWOB and acanthosis nigricans prevalence in young children after the intervention [5]. One component of the multi-level CHL intervention was the implementation of Food Friends^®^: Fun with New Foods™ and Get Movin’ with Mighty Moves™ curricula, referred to as “Food Friends and Mighty Moves” or FFMM, in childcare centers [6,7]. Although the outcomes of FFMM were not evaluated independently, the feasibility and implementation in Guam expanded the reach into preschool programs that provide school readiness support and family engagement.

FFMM aims to improve diet quality, such as increasing fruits and vegetables (FV) and physical activity in early childhood by addressing food neophobia and developing gross motor skills, respectively [8,9]. There are limited diet data among young children in Guam, yet one study revealed the mean intake of FV among 2–8-year-olds was 0.88 and 0.61 cups per day, respectively, which does not meet the recommendations [3]. Additionally, the majority of young children exceeded the recommended, 2 h or less, for screen-time with a mean duration of 5.29 h per day [3], which consequently decreases the chance to be physically active. 

Early childhood interventions show a great potential to promote the development of long-term healthful habits, such as regular physical activity and healthy eating to prevent obesity [10,11,12]. One study in Guam found children’s willingness to try FV improved after receiving nutrition education [13]. In addition to knowledge, preference is another personal factor that determines children’s FV intake. Early food likes and dislikes are influenced by preferences, yet modifiable through repeated exposures to novel and disliked foods in a positive, supportive environment [14,15]. Despite the growing body of childhood obesity research, there is still a lack of research including indigenous young children. Guam is a US territory located in the northwestern Pacific region of Micronesia and the southernmost island of the Mariana Islands where 37% of the population are CHamoru, the indigenous people of Guam; 12% are other Pacific islanders, 26% are Filipino and 7% are other Asian [16].

The Food Friends^®^: Fun with New Foods™ and Get Movin’ with Mighty Moves™, or FFMM, are research-based nutrition and physical activity curricula developed by researchers in Colorado State University. During the CHL program, FFMM was implemented in childcare centers without adaptation and evaluation. For this study, we will examine the effectiveness of the FFMM curricula on willingness to try fruits and vegetables (FV) and gross motor (GM) skills among preschool children in Guam during two school years (i.e., SY2017–2018 and SY2018–2019) as part of a research project to determine the best practices for obesity prevention in Guam. 

## 2. Materials and Methods

### 2.1. Study Design

This study was a pre-post community-based study design targeting preschool children, 3–5 years old, from three (3) Guam Department of Education (GDOE) preschool programs: Guam Head Start (HS) Program, gifted and talented education pre-Kindergarten (Pre-GATE), and Pre-Kindergarten (Pre-K). “Head Start” is a comprehensive program funded by the U.S. Department of Health & Human Services that promotes school readiness of preschool children, 3–5 years, from low-income families by enhancing their cognitive, social, and emotional development. HS supports children’s growth and development in a positive learning environment through comprehensive services in the areas of education and child development, health, and family and community engagement. Locally, the Guam Department of Education is the grantee for Head Start. The GATE pre-K program, referred to as Pre-GATE, implements a curriculum specifically designed for 4 year old gifted children that includes acceleration and enrichment activities to ensure their physical, social, emotional, and intellectual needs are met without pressure and unnecessary structure. The Pre-Kindergarten (Pre-K) program follows the Guam Early Learning Guidelines for young children 3–5 years that focuses on five areas—physical development, health and safety; self-concept and social-emotional development; cognitive development; communication, language development and literacy, and creative development.

During SY2017–2018, preschool programs that were prepared and willing to implement the Food Friends^®^: Fun with New Foods and Get Movin’ with Mighty Moves™ (FFMM) curricula were pre-K (4 classrooms) and Guam HS (7 classrooms). Other preschool programs that participated in this study in SY2017–2018, were half-day Guam HS sites (7 classrooms) and the pre-GATE program (4 classrooms) from the same villages that did not receive FFMM. For the Guam HS Program a standard curriculum implemented in all HS classrooms was “I Am Moving, I Am Learning (IMIL)”, which has similar components to FFMM. IMIL was a curriculum used in previous years for all Guam Head Start Program classrooms which includes a flexible framework of strategies to promote movement skills, healthy eating, parent engagement, and a healthy workplace and community [17]. Among the Guam HS Program: (1) classrooms that were transitioning to full-day schedules and, therefore, had the need for additional classroom activities implemented FFMM plus IMIL (classified for this study as “Enhanced Intervention”); (2) classrooms that had a half-day schedule implemented only IMIL (classified for this study as “Standard”). Pre-K program classrooms that only implemented FFMM were classified as “Intervention” and Pre-GATE program classrooms that did not receive either FFMM or IMIL were the “Control”. During SY2018–2019, all HS classrooms (i.e., half-day and full-day) implemented both FFMM and IMIL (classified for this study as “Enhanced Intervention”) and Pre-GATE and Pre-K implemented FFMM only (considered for this study as “Intervention”). 

Child participants were recruited from study sites (i.e., preschool classrooms) during orientation or the first week of school. Research staff conducted study presentations at orientation or distributed recruitment packets (e.g., recruitment flyer and study forms) to parents. Parents provided consent for their child (ren) to participate and completed the About My Child (i.e., age, sex, ethnicity/race) and healthy behavior forms. The data from the behavior forms were not used in this study. Child assent was obtained for pre- and post-assessments. Child participants were provided a USD 10 gift card after completing each assessment. The University of Guam (UOG) Institutional Review Board (CHRS 17-139) and Guam Department of Education approved the study protocols. 

### 2.2. Study Participants

There were a total of 316 children recruited from all sites during SY2017–2018, where 110 received the enhanced intervention, 63 received the intervention, 92 received the standard, and 51 were in the control group. All study sites (i.e., enhanced intervention, intervention, standard, and control) received the FFMM curricula the following year, SY2018–2019, where 355 children participated in the study activities, specifically the pre- and post-assessments. Notably, all children received FFMM in intervention sites no matter if they participated in the study or not in both study years.

### 2.3. Intervention

The intervention was implemented by trained teachers and teachers’ aides working within the study sites (i.e., preschool classrooms). FFMM teacher training was conducted prior to classroom implementation by one of the FFMM developers and/or trained research staff. A major component of the curricula are the eight (8) Food Friends (stuffed puppets) characters that are incorporated in lesson activities and featured in lesson materials for both children and parents [18], which was a component of training. Food service provider training was also conducted by trained research staff prior to the implementation of the FF on food preparation and delivery schedule to support taste tests. 

The Food Friends^®^: Fun with New Foods™ (FF) curriculum was implemented in September until December of each school year. Teachers preferred to implement FF a few weeks after the start of the school year for preschoolers to acclimate. The FF intervention program lasted 12 weeks, with two lessons per week, for a total of 24 lessons. Each week children participated in hands-on food and nutrition activities, story time, and/or taste tests that lasted about 15–20 min. Children were given repeated exposures to the same new foods (i.e., Gouda cheese and raw daikon radish) for eight weeks followed by weekly opportunities to try new foods (foods varied based on seasonality/availability) [8]. During these months, lesson foods were most available, which also informed the FF curriculum implementation timeline.

The Get Movin’ with Mighty Moves™ (MM) curriculum was implemented each school year from January to May, which was ideal for implementing all 18-weeks of the curriculum. Teachers had up to four (4) lessons per week to select from and were asked to implement at least two (2) lessons each week, for a total of 36–72 lessons. Children participated in activities that focused on one of the gross motor skill categories each week: stability (e.g., trunk strength), locomotor (e.g., running, hopping, skipping), or manipulation (e.g., ball skills) and described in detail elsewhere [9]. 

### 2.4. Evaluations

Data collection was conducted at each study site before and after the implementation of both curricula, FFMM, which was within two weeks after the start and before the end of the school year, respectively. This aligned with program assessments that are a regular part of each preschool program. Study assessments did not take away from instructional time and did not appear to be different from ongoing school day activities. 

Children’s willingness to try new foods and FV were assessed using the validated *Adapted WillTry* tool for children 3–11 years in Guam [13,19,20]. Only FV data will be reported in this study, which were captured in one of four FV scales in the *Adapted WillTry* tool: local novel (6 items), local common (4 items), imported (3 items), and total FV (14 items) [19]. Trained research staff conducted one-to-one interviews with children, where children self-reported their willingness to try new foods and FV. These methods have been tested in a similar population in Guam and described elsewhere [13].

Gross motor skills were observed and recorded using the Get Movin’ with Mighty Moves™ Pre- and Post- Program Evaluation Tool and Guidelines (provided with the curriculum) [9], which consists of five (5) items assessing: (1) standing on one foot (dominant and non-dominant leg), (2) standing on tiptoes, (3) walking line backward, (4) tossing ball underhand (distance), and (5) tossing ball with or without opposition. Each gross motor skill was assigned a score to determine proficiency (i.e., 1) or levels (i.e., 1, 2, or 3) based on the age-appropriate criteria outlined in the Get Movin’ with Mighty Moves™ Pre- and Post- Program Evaluation Tool and referenced in standard assessment tools used in Guam preschool programs, such as Brigance^®^ Early Childhood Screen III and Developmental Indicators for the Assessment of Learning (DIAL-4). Standing on one foot (dominant) and the other (non-dominant) was given a score of “1” if held for 5 s or more for 3-year-olds and 10 s or more for 4- and 5-year-olds. Similarly, “1” was scored if a child could stand on tiptoes for 4 s or more for 3-year-olds or 8 s or more for 4- and 5-year-olds. Durations less than the criteria for these skills were assigned a score of “0”. All children were scored “1” for less than 2 steps, “2” for 2–4 steps, and “3” for 5 steps or more when they were observed walking line backward (toe-to-heel). Any child that tossed the ball underhand more than 10-feet was scored with “1” and less than 10-feet was scored “0”. While observing the same child toss the ball underhand, research staff also observed whether the child used opposition “1” or no opposition “0”. The Pre- and Post-Program Evaluation Tool describes opposition as tossing the ball underhand, rotating upper body, moving arms in opposition to legs, and beginning toss by moving arms down and back.

### 2.5. Data Analysis

All data collection forms were created in Qualtrics and used for data entry, then exported as an Excel file that was imported into SPSS (version 27). Double-data entry procedures were used. Two-way mixed ANOVAs were used to examine between (group) and within subjects (pre- and post-assessment) program effects for all *Adapted WillTry* FV scores and both study years. For SY2017–2018, groups were defined as enhanced intervention (i.e., FFMM and IMIL), intervention (i.e., FFMM only), standard (i.e., IMIL only), and control. For SY2018–2019, intervention groups were categorized as enhanced intervention (i.e., Head Start) and intervention (i.e., pre-GATE and pre-K). Simple main effect analyses were conducted when ANOVA revealed significant interactions. The Bonferroni correction was used to adjust for multiple comparisons. *Adapted WillTry* FV pre- and post-means with standard deviations (SDs) were reported for local novel, local common, imported categories, and total FV scales.

Once proficiency was calculated for each gross motor skill the number of children that were proficient in each skill was calculated and reported in percent for each study year, except walking line backward for which means were reported. An exact McNemar’s test was used to examine the difference in the proportion of children proficient in each skill pre- and post-intervention. A Wilcoxon test was conducted to determine the effect of the intervention on walking line backward performance. Multiple logistic regression was used to examine the differences in post-percentages between groups and adjusted for pre-percentages, sex, age and ethnicity. *p*-values < 0.05 were considered statistically significant.

## 3. Results

Demographic characteristics of the child participants are presented in Table 1. The majority of children were CHamoru or Filipino and 4–6 years old in both study years. 

Four (4) individual two-way mixed ANOVAs were performed for each school year separately. The *Adapted WillTry* FV scores for local novel, local common, imported, and total FV were the dependent variables. In SY2017–2018, the interaction between time and group were not significant for local novel, local common, and total *Adapted WillTry* FV scores, but there was a significant interaction between time and groups on the imported *Adapted WillTry* FV score. Post-hoc test using the Bonferroni correction revealed that the intervention group had a significant increase in imported *Adapted WillTry* FV score when compared with the other three groups (Table 2).

In SY2018–2019, there was a significant interaction between the group and time on all *Adapted WillTry* FV scores except imported *Adapted WillTry* FV. The intervention group (i.e., non-HS) had significantly lower total, local novel and local common *Adapted WillTry* pre-scores than the enhanced intervention group. After completing FFMM, the intervention group showed a significant increase for all *Adapted WillTry* FV scores. The mean *Adapted WillTry* FV scores did not change over time in the enhanced intervention group and both groups had similar post-scores (Table 2). 

For SY2017–2018, it was hypothesized that the intervention groups (i.e., enhanced intervention and intervention) would have higher levels of proficiency for all motor skills than other groups. To examine this hypothesis, multiple logistic regression of the post-scores for each motor skill were conducted. There were no significant differences (*p* < 0.05) between any of the groups for all gross motor skills post-scores, even after adjusting for sex, age, ethnicity and pre-scores. Within-group comparisons of changes in skills over time were examined using McNemar’s and Wilcoxon tests. The enhanced intervention group was the only one that improved all stability gross motor skills (*p* < 0.05), that is standing on one leg (either leg) and standing on tiptoes. The intervention group significantly increased two stability gross motor skills (*p* < 0.05) and significantly decreased one gross motor skill (tossing ball underhand—opposition). The standard group only improved one stability gross motor skill and the control group improved in two stability gross motor skills. None of the groups improved in tossing a ball (distance) (Table 3). 

In SY2018–2019 no significant differences were found in all gross motor skills post-scores between the two groups even after adjusting for sex, age, ethnicity and pre-scores. Within-group analyses revealed a significant improvement in all GM skills (*p* < 0.05), except tossing ball underhand (opposition) in the enhanced intervention group, and tossing ball underhand (distance) and tossing a ball underhand (opposition) in the intervention group (Table 4). 

## 4. Discussion

In SY2017–2018, there were differences in *Adapted WillTry* FV scores. The intervention group had a significant increase in imported *Adapted WillTry* FV scores when compared with the other three groups. No significant differences between groups were found on the other *Adapted WillTry* FV scores. The post-score of imported *Adapted WillTry* FV in the intervention group was significantly higher than the pre-score. Since the enhanced intervention (i.e., HS) was composed of both the FFMM and the IMIL curricula, it was expected that this group would demonstrate greater improvements in the *Adapted WillTry* FV scores compared to the intervention group; however, this was not the case. The enhanced intervention group actually demonstrated a decrease, although not significant, on all FV scores. The reason for this is unknown, but may be due to the enhanced intervention teachers focusing on implementing their required curriculum, IMIL, and only teaching the FFMM when time allowed. In addition, the intervention group had significantly lower *Adapted WillTry* FV scores (all *p* ≤ 0.01) at pre-assessment compared to the enhanced intervention group (i.e., HS). Therefore, FFMM may be more effective in improving willingness to try FV among children that initially have a low willingness to try FV (score). The community-based study design may have also contributed to the lack significant improvements of *Adapted WillTry* FV scores among children who received the enhanced intention in SY2017–2018. FFMM was implemented in the Pre-K program (four classrooms) and only full-day classrooms in the Guam Head Start Program (seven classrooms), which was also the first year (SY2017–2018) to pilot a full-day class schedule in the Guam Head Start Program. To fulfill the day’s activities, full-day classrooms continued to implement the curriculum, “I Am Moving, I Am Learning” (IMIL) in addition to implementing the FFMM intervention. Similarly, the Guam Head Start Program half-day classrooms implemented IMIL that has similar components to the FFMM intervention. The effect of the FFMM intervention on willingness to try FV may have been attenuated by the implementation of IMIL in both HS groups. 

As mentioned previously, the intervention only demonstrated a significant improvements in imported *Adapted WillTry* FV scores in SY2017–2018. This may be due to several factors. First, the post- *Adapted WillTry* tool was administered at the end of the SY, 5 months after the FF curriculum ended. Therefore, children may have forgotten some of the local common and local novel foods they had been introduced to earlier in the school year. Second, the FFMM curriculum may need to be modified so that it is more culturally relevant and better promotes local FV. Third, previous studies have shown that there is an abundance of imported and processed foods on Guam [21], and a majority of Guam residents consume a high volume of processed foods that are imported [3,22]. Thus, imported foods, including FV, are acceptable and possibly even desirable to Guam residents, including children. The abundance, acceptability, and easy access to imported foods on Guam may have contributed to higher imported *Adapted WillTry* FV scores, especially if the FFML curriculum promoted mainly imported FV.

In SY2017–2018, there were no significant differences in gross motor skills found between groups. The group that had a greater increase in gross motor skills was the enhanced intervention group (i.e., Guam Head Start program), with a significant improvement on all stability gross motor skills. This was expected as the enhanced intervention group was exposed to both the FFML and the IMIL curricula. Unfortunately, authors are uncertain as to the exact number of lessons given to each group as teachers were inconsistent in their intervention fidelity reports. However, having both IMIL and/or FFMM positively influenced gross motor skill development.

In SY2018–2019, the intervention group reported significant increases in all *Adapted WillTry* FV scores except imported FV when compared with the enhanced intervention group (i.e., Guam Head Start program). All *Adapted WillTry* FV scores improved from pre- to post- in the intervention group. The children in the enhanced intervention group may have had a higher willingness to try (score) at pre-assessment related to the family style mealtimes during the school day which supports FV intake [23]. For the enhanced intervention group, most *Adapted WillTry* FV scores were in the expected direction, yet the increase was not significant. Being that the FFMM curricula was developed in Colorado and used food and food characters (stuffed puppets) known to this state (e.g., hamburger puppet), curricula modifications to include local foods and cultural components to be more relevant to Guam residents, predominantly CHamorus and Filipinos, are warranted. Regarding gross motor skills, no significant differences were found between groups during their progression over the school year. Stability gross motor skills improved in both groups and the enhanced intervention group also improved in tossing the ball–underhand (distance) and walking the line backwards.

The actual child eating behavior of trying new foods has been related to children who perceive themselves as more willing to try [24]. In this study children’s self-competence to try new foods was assessed immediately followed by observations of willingness to taste novel foods. Although *Adapted WillTry* FV post-scores were assessed approximately 5 months after the FF curriculum ended, which was at the end of the school year after the MM curriculum was completed, the mean *Adapted WillTry* scores for all the FV scales maintained a trend (from high to low) in willingness to try imported, local common, and local novel FV observed in previous studies [11,19]. This was observed for pre- and post-assessment mean scores and for all groups in both study years, demonstrating the robustness of the *Adapted WillTry* tool and FV scales; and indicating that preschool children in Guam are more likely to try (eat) imported FV over local FV, which further justifies the need for a culturally relevant curriculum to promote local FV.

Although the community-based study design may have influenced the study outcomes, this study included new and long-standing preschool programs in Guam that, for the first time, implemented the same curricula in a unified approach to reach young children during SY2018–2019. This study also demonstrated the sustainability of one of the components of the multi-level CHL intervention, and further demonstrating the feasibility and implementation of FFMM in Guam. None of the study activities, including FFMM implementation and assessments, interrupted the regular school day activities. The food tasting activities complemented the family style mealtimes in the Head Start program and were the only food-related activity in the classroom for Pre-GATE and Pre-K. The FFMM curricula addressed the Pre-Kindergarten Curriculum Standards in the areas of health, physical education, fine arts, language arts/reading, math, social science, and science. Overall, this study helps to fill a research gap in the Pacific region as diet and physical activity is not well documented in indigenous peoples, such as CHamorus and other Pacific Islanders, especially young children.

This study is not without limitations. Researchers and staff asked teachers to complete fidelity logs to document the number of lessons completed; however, not all teachers completed or submitted logs. We were unable to assess lesson dose and compare groups. Due to the nature of this community-based study, the number of lessons likely varied related to unplanned events, such as fire drills or seasonal weather disturbances (e.g., tropical depressions). Similarly, restricting the type of activities that took place in the classroom was not possible with regard to lesson plans and/or curriculum. The Guam Head Start Program is required to address health and wellness in the school-day and used IMIL when FFMM was not implemented, such as in the standard group during SY2017–2018. Another limitation was attrition—not all students completed all assessment periods due to being absent or refusal related to competing activities (e.g., children playing, class activity).

## 5. Conclusions

In SY2017–2018, no differences in GM skills were found among groups; however, there was an improvement in three of six GM skills in enhanced intervention and intervention groups. There was an increase in the willingness to try imported FV in the intervention group. In SY2018–2019, four of six GM skills significantly improved for both groups. Willingness to try FV only improved for children participating in non-HS programs. FFMM adaptations/modifications are needed to be more culturally relevant to Guam, especially in HS programs.

## Figures and Tables

**Table 1:** Characteristics of child study participants in Guam preschool programs that received FFMM, IMIL, both, or none during two school years (i.e., SY2017–2018 and SY2018–2019).

Child Characteristics	SY2017–2018 (*n* = 316)				SY2018–2019 (*n* = 355)	
	Enhanced Intervention ^1^	Intervention ^2^	Standard ^3^	Control ^4^	Enhanced Intervention ^1^	Intervention ^2^
Sex ^a^						
Female	51 (46.4)	26 (41.3)	44 (47.8)	29 (56.9)	102 (43.8)	63 (51.6)
Male	59 (53.6)	37 (58.7)	48 (52.2)	22 (43.1)	131 (56.2)	59 (48.4)
Age ^1^ (years)						
2–3	11 (11.1)	0 (0.0)	18 (22.5)	0 (0.0)	35 (15.6)	2 (1.7)
4–6	88 (88.9)	60 (100.0)	62 (77.5)	51 (100.0)	189 (84.4)	116 (98.3)
Ethnicity ^a^						
CHamoru	44 (40.0)	21 (33.9)	56 (61.5)	15 (29.4)	117 (50.0)	31 (27.2)
Filipino	27 (24.5)	23 (37.1)	14 (15.4)	20 (39.2)	40 (17.1)	38 (33.3)
Other Asian	2 (1.8)	7 (11.3)	2 (2.2)	4 (7.8)	9 (3.8)	10 (8.8)
Other Pacific Islander	22 (20.0)	1 (1.6)	12 (13.2)	1 (2.0)	51 (21.8)	5 (4.4)
2+ race/ethnic groups and other ^b^	15 (13.6)	10 (16.1)	7 (7.7)	11 (21.6)	17 (7.3)	30 (26.3)

^1^ Preschool children received Food Friends^®^: Fun with New Foods and Get Movin’ with Mighty Moves™ (FFMM) and I am Moving, I am Learning (IMIL) lessons. ^2^ Preschool children received Food Friends^®^: Fun with New Foods and Get Movin’ with Mighty Moves™ (FFMM) lessons. ^3^ Preschool children received I am Moving, I am Learning (IMIL) lessons. ^4^ Preschool children with no intervention. ^a^ Not all parents reported child participant characteristics; therefore, *n* is different for sex, age group, and ethnicity. ^b^ Child participants that identified as Black, White, American Indian or Alaska Native or with two (2) or more race/ethnic groups.

**Table 2:** Means, standard deviations (SD), and mixed ANOVAs interaction effects between time and group for all Adapted WillTry fruit and vegetable (FV) pre- and post-scores by study group in both school years ^a^.

SY2017–2018										
Adapted WillTry FV Scales	Enhanced Intervention ^1^		Intervention ^2^		Standard ^3^		Control ^4^		*p*	η^2^_p_
	Pre	Post	Pre	Prost	Pre	Post	Pre	Post		
	Mean ± SD		Mean ± SD		Mean ± SD		Mean ± SD			
Total FV	2.52 ± 0.69	2.40 ± 0.66	2.16 ± 0.74	2.31 ± 0.56	2.46 ± 0.66	2.40 ± 0.64	2.3 ± 0.72	2.20 ± 0.61	0.220	0.023
Local Novel	2.45 ± 0.78	2.25 ± 0.75	2.04 ± 0.83	2.16 ± 0.66	2.36 ± 0.78	2.21 ± 0.79	2.17 ± 0.85	2.06 ± 0.77	0.242	0.020
Local Common	2.47± 0.75	2.42 ± 0.71	2.21 ± 0.77	2.25 ± 0.67	2.34 ± 0.77	2.36 ± 0.74	2.37 ± 0.75	2.19 ± 0.76	0.517	0.011
Imported	2.72 ± 0.52	2.68 ± 0.56	2.41 ± 0.73	2.75 ± 0.5 ^a^	2.74 ± 0.44	2.70 ± 0.5	2.62 ± 0.53	2.50 ± 0.53	0.001 ^b^	0.072
**SY2018–2019**										
	**Enhanced** **Intervention ^1^**				**Intervention ^2^**				***p***	**η^2^_p_**
	**Pre**		**Post**		**Pre**		**Post**			
	**Mean ± SD**				**Mean ± SD**					
Total FV	2.49 ± 0.63		2.49 ± 0.55		2.22 ± 0.61		2.47 ± 0.54 ^a^		0.012	0.029
Local Novel	2.41 ± 0.72		2.37 ± 0.68		2.03 ± 0.76		2.31 ± 0.72 ^a^		0.011	0.030
Local common	2.49 ± 0.73		2.51 ± 0.63		2.29 ± 0.68		2.52 ± 0.55 ^a^		0.041	0.019
Imported	2.67 ± 0.54		2.72 ± 0.49		2.62 ± 0.47		2.79 ± 0.37 ^a,b^		0.131	0.011

*p*: *p*-value of the time x group interaction effect determined by two-way mixed ANOVA. η^2^_p_: partial eta squared. ^1^ Preschool children received Food Friends^®^: Fun with New Foods and Get Movin’ with Mighty Moves™ (FFMM) and I am Moving, I am Learning (IMIL) lessons. ^2^ Preschool children received Food Friends^®^: Fun with New Foods and Get Movin’ with Mighty Moves™ (FFMM) lessons. ^3^ Preschool children received I am Moving, I am Learning (IMIL) lessons. ^4^ Preschool children with no intervention. ^a^ *p* < 0.05 versus pre-score. ^b^ The intervention group had a significant increase on imported FV values when compared with the other 3 groups as determined by Bonferroni test.

**Table 3:** Percent of preschool child participants that met gross motor (GM) skill proficiency and mean scale score for walking line backgrounds at pre- and post-study period during school year (SY) 2017–2018.

	SY2017–2018											
	Enhanced Intervention ^1^ (*n* = 53)			Intervention ^2^ (*n* = 48)			Standard ^3^ (*n* = 45)			Control (*n* = 33)		
Gross Motor Skill	Pre	Post	*p*-Value	Pre	Post	*p*-Value	Pre	Post	*p*-Value	Pre	Post	*p*-Value
	(%)			(%)			(%)			(%)		
Standing on 1 foot (dominant Leg)	34.0	58.5	0.011 ^a^	41.7	66.7	0.017 ^a^	37.8	60.0	0.031 ^a^	39.4	63.6	0.057 ^a^
Standing on 1 foot (non-dominant leg)	30.2	58.5	0.001 ^a^	35.4	62.5	0.021 ^a^	35.6	51.1	0.143 ^a^	33.3	57.6	0.039 ^a^
Standing on tiptoes	30.2	56.6	0.014 ^a^	45.8	62.5	0.152 ^a^	42.2	60.0	0.115 ^a^	42.2	81.8	<0.001 ^a^
Tossing ball-underhand (distance)	37.5	36.8	>0.999 ^a^	48.0	30.0	0.093^a^	45.5	48.9	>0.999 ^a^	37.5	33.3	>0.999 ^a^
Tossing ball-underhand (opposition)	83.8	73.7	0.18 ^a^	96.0	66.0	<0.001 ^a^	88.9	75.6	0.146 ^a^	87.5	72.7	0.180 ^a^
	Means			Means			Means			Means		
Walking line backward (toe-to-heel) ^b^	2.21	1.96	0.062 ^b^	2.18	2.08	0.539 ^b^	2.02	1.8	0.174 ^b^	2.09	2.13	0.875 ^b^

^1^ Preschool children received Food Friends^®^: Fun with New Foods and Get Movin’ with Mighty Moves™ (FFMM) and I am Moving, I am Learning (IMIL) lessons. ^2^ Preschool children received Food Friends^®^: Fun with New Foods and Get Movin’ with Mighty Moves™ (FFMM) lessons. ^3^ Preschool children received I am Moving, I am Learning (IMIL) lessons. ^a^ Based on McNemar’s test. ^b^ Means for the scale is reported. *p*-values are based on Wilcoxon test.

**Table 4:** Percent of preschool child participants that met gross motor (GM) skill proficiency and mean scale score for walking line backgrounds at pre- and post-intervention during school year (SY) 2018–2019.

	SY2018–2019					
	Enhanced Intervention ^1^			Intervention ^2^		
	(*n* = 150 to 162)			(*n* = 93 to 104)		
Gross Motor Skill	Pre	Post	*p*-Value	Pre	Post	*p*-Value
	(%)			(%)		
Standing on 1 foot (dominant leg)	37.0	56.0	0.001 ^a^	50.0	66.7	0.006 ^a^
Standing on 1 foot (non-dominant leg)	24.1	50.0	<0.001 ^a^	34.0	55.9	0.002 ^a^
Standing on tiptoes	34.6	55.0	<0.001 ^a^	35.0	57.0	0.001 ^a^
Tossing ball-underhand (distance)	39.3	58.0	0.008 ^a^	37.5	44.7	0.201 ^a^
Tossing ball-underhand (opposition)	61.8	65.8	0.457 ^a^	61.5	67.0	0.243 ^a^
	Means		*p*-value	Means		*p*-value
Walking line backward (toe-to-heel) ^b^	1.56	1.78	0.008 ^b^	1.60	1.98	0.018 ^b^

^1^ Preschool children received Food Friends^®^: Fun with New Foods and Get Movin’ with Mighty Moves™ (FFMM) and I am Moving, I am Learning (IMIL) lessons. ^2^ Preschool children received Food Friends^®^: Fun with New Foods and Get Movin’ with Mighty Moves™ (FFMM) lessons. ^a^ Based on McNemar’s test. ^b^ Means for the scale reported. *p*-values are based on Wilcoxon test.

## Data Availability

The data presented in this study are available on request from the corresponding author. The data are not publicly available due to privacy restrictions.
